# CDK4/6 inhibitors: A potential therapeutic approach for triple negative breast cancer

**DOI:** 10.1002/mco2.97

**Published:** 2021-11-17

**Authors:** Lubaid Saleh, Caroline Wilson, Ingunn Holen

**Affiliations:** ^1^ Department of Oncology and Metabolism Medical School University of Sheffield Sheffield UK; ^2^ Weston Park Hospital Whitham Road Sheffield UK

**Keywords:** CDK/4/6 inhibitors, metastatic, triple negative breast cancer

## Abstract

Triple negative breast cancer (TNBC) cells lack expression of the estrogen receptor (ER), progesterone receptor (PR), and human epidermal growth factor receptor‐2 (HER‐2). Thus, TNBC does not respond to hormone‐based therapy. TNBC is also an aggressive subtype associated with poorer prognoses compared to other breast cancers. Conventional chemotherapeutics are used to manage TNBC although systemic relapse is common with limited benefits being reported as well as adverse events being documented. Here, we discuss current therapies for TNBC in the neo‐ and adjuvant settings, as well as recent advancements in the targeting of PD‐L1‐positive tumors and inclusion of PARP inhibitors for TNBC patients with BRCA mutations. The recent development of cyclin‐dependent kinase (CDK) 4/6 inhibitors in ER‐positive breast cancers has demonstrated significant improvements in progression free survival in patients. Here, we review preclinical data of CDK 4/6 inhibitors and describe current clinical trials assessing these in TNBC disease.

## Graphical abstract

Triple negative breast cancer (TNBC) is an aggressive subtype associated with poorer prognoses compared to other breast cancers. Conventional chemotherapeutics are used to manage TNBC although systemic relapse is common with limited benefits being reported. The identification of alternative pathways important for the survival of cancer cells has led to the development of CDK4/6 inhibitors that have shown great promise in preliminary clinical trials in TNBC patients, in the presence of chemotherapy and alone as monotherapy. With early preclinical studies further novel agents have been identified that may lead to new strategies in treating TNBC and providing improved prognoses for patients.

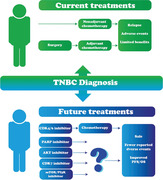

## 1 INTRODUCTION

Breast cancer (BC) remains the most common cause of death in women, resulting in over half a million deaths annually worldwide.^1^ BC falls into four main subtypes, and the most common are luminal A (LBC‐A) and luminal B (LBC‐B) that represent 60% (40% and 20%, respectively) of cases. The more aggressive human epidermal growth factor receptor‐2 (HER‐2)‐positive and triple negative (lacking ER/PR and HER‐2) BC (TNBC) subtypes account for approximately 25% and 15% of cases, respectively. Each subtype is characterized by prognostic markers that can be identified by molecular profiling.^2^ LBC‐A is positive for the estrogen receptor (ER) and/or progesterone receptor (PR) receptors but negative for HER‐2. Similarly, LBC‐B is ER and/or PR positive but can also be HER‐2 positive or negative. Thus, LBC‐B has a higher rate of recurrence and poorer prognosis compared to LBC‐A. Growth of ER‐/PR‐positive BCs is heavily dependent on the expression of these receptors as they stimulate signaling pathways involved in cellular processes such as proliferation, apoptosis, and angiogenesis.^3^ This dependence on ER/PR signaling has allowed for the development of endocrine therapies that target these receptors and their downstream pathways, such as ER blockers (often known as selective ER modulators [*SERMs*]), aromatase inhibitors (*AIs*), and ER downregulators (selective ER downregulators [*SERDs*]). Tamoxifen (SERM), letrozole (AI), and fulvestrant (SERD) are examples of endocrine therapies that are widely used in ER‐positive BC, often recommended to patients for a 5‐ or 10‐year period after breast surgery with significant improved outcomes.^4^, ^5^ In HER‐2‐positive BC, overexpression of the HER‐2 receptor has served as a suitable target for novel agents. For example, the monoclonal antibodies, trastuzumab and pertuzumab, cause downregulation and inhibition of the HER‐2 and HER‐1 receptors, respectively, resulting in improved survival outcomes.^6^ Additionally, in HER‐2‐positive BC, the antibody‐conjugated agents TDM1 (trastuzumab conjugated to the cytotoxic agent DM1) and T‐Dxd (trastuzumab deruxtecan) have shown significant antitumor activity and are used as standard therapy in early and advanced disease, respectively.^7^, ^8^, ^9^, ^10^ In contrast, TNBC accounts for 170,000 cases annually worldwide in which patients have poor prognosis and overall survival (OS) as well as higher distant recurrence rates.^11^, ^12^ Although initially responsive to chemotherapy, TNBC patients have relatively short disease‐free survival (DFS) rates compared to those with hormone‐receptor‐positive disease.^13^ Coupled with the lack of targetable receptors, effective treatment remains a challenge for TNBC patients with novel agents urgently needed to improve outcome.

## 2 TNBC PATHOLOGY AND METASTASIS

**FIGURE 1:**
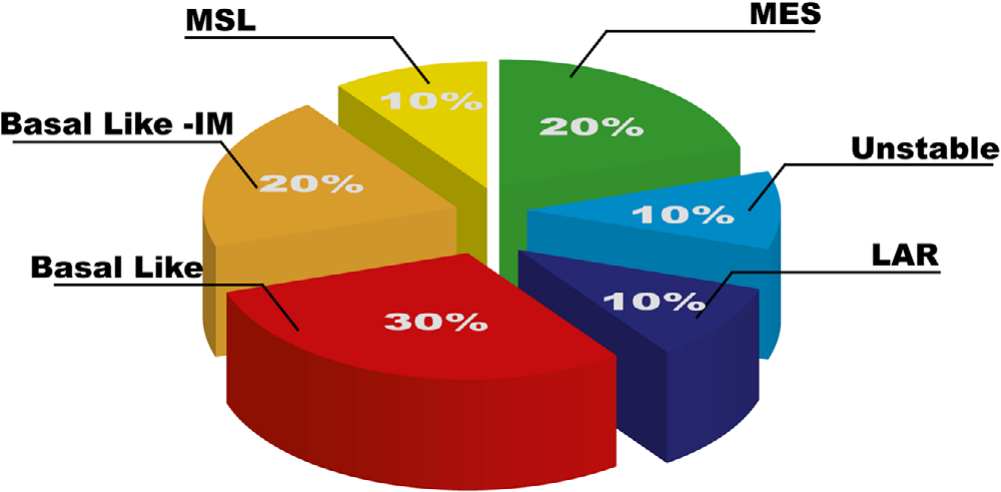
Proportion of TNBC subtypes. The unstable subtype is characterized by its cellular proliferation and responses to DNA damage Abbreviations: IM, immunomodulatory; LAR, luminal androgen receptor; MES, mesenchymal; MSL, mesenchymal stem like.

TNBC falls into four main subtypes identified by early gene profiling studies and histological analyses; basal‐like, luminal (androgen receptor [AR]) like (LAR), mesenchymal (MES), and mesenchymal stem like (MSL). Although the terms basal‐like BC and TNBC are often used interchangeably, they are not the same. TNBC is a description of the immunophenotype of BC that is negative for ER, PR, and HER‐2, whereas basal‐like BC describes the molecular phenotype, with approximately 50% of TNBC falling within this subtype (Figure 1).^14^ Basal‐like BC, first discovered by first generation cDNA microarrays,^15^ is characterized by enriched cell cycle and cell division pathways, as well as elevated DNA damage response pathways.^14^, ^16^ Additionally, immune suppressed basal‐like (IM) subtypes, identified by gene profiling, are enriched for factors involved in immune cell signaling (basal‐like immune activated [BLIA]), resulting in a favorable prognosis despite its association with a high‐grade histology.^17^ Burstein et al. also described an IM subtype (basal‐like immune suppressed [BLIS]) that exhibited low expression of immune cell differentiation and immune signaling and is associated with poor prognosis as demonstrated by low DFS rates.^17^ The LAR subtype is characterized by its overexpression of the AR, 10‐fold greater than the other subtypes.^18^ Although ER negative, LAR BC cells may express *ESR1* (gene encoding ER) and other estrogen‐regulated genes, as well as pathways that regulate steroid synthesis, porphyrin metabolism, and androgen/estrogen metabolism.^14^ The MES and MSL subtypes are heavily enriched in mechanisms involved in cell motility and extracellular receptor interaction and cell differentiation.^14^, ^16^ More recent analyses of breast tumors obtained from the molecular taxonomy of BC international consortium (METABRIC) identified 10 subtypes that were associated with histological type, tumor grade, receptor status, and lymphocytic infiltration.^19^ Further analysis of the METABRIC and The Cancer Genome Atlas (TCGA) databases using copy number variants (CNVs) of the different subtypes proved to be an accurate method for the diagnosis of BCs compared to mRNA biomarkers.^20^ These biomarkers, and their associated signaling pathways, provide targetable opportunities for therapeutic agents in TNBC disease.

Identification of these various subtypes is important for determining the most suitable therapeutic approaches for TNBC patients. In a retrospective study of early BC, investigators revalidated existing gene expression microarray data from 146 TNBC patients, 130 of which had received standard neoadjuvant chemotherapy with evaluable pathologic response data. The authors were then able to classify the TNBC subtypes and correlate them to the pathological complete response (pCR) status.^21^ They found that TNBC subtype was significantly associated with pCR status. For example, the basal‐like1 (BL1) subtype was associated with the highest pCR rate (52%), whereas the basal‐like2 (BL2) and LAR had the lowest pCRs (0% and 10%, respectively).^21^ Though specific tumor subtypes can be a predictor of pCR, further classification and understanding of these subtypes may help direct personalized therapeutic strategies for TNBC patients who have currently incurable metastatic disease.

### 2.1 Metastatic TNBC

Metastasis in BC is a complex multistep process that involves the infiltration of tumor cells into the surrounding tissue followed by transendothelial migration into blood vessels (intravasation) and subsequently extravasation into distant sites.^22^ Metastasis may also occur through the lymphatic system into the lymph nodes; thus, tumor‐positive lymph nodes are important predictors of tumor aggressiveness for most BCs.^23^ More specifically, in metastatic TNBC (mTNBC), a higher rate of node positivity is observed with visceral metastasis more likely to occur in the lungs and brain.^24^, ^25^ In addition, TNBC patients with visceral metastases demonstrate shorter median survival rates compared to non‐TNBC with limited response to chemotherapy.^26^, ^27^

## 3 CURRENT TREATMENTS FOR TNBC

Due to the biologically aggressive nature of TNBC, prognosis is very poor (median OS being 10.2 months with a 5‐year survival rate of 65% for localized tumors and 11% that have spread to distal organs^28^, ^29^) despite patients sometimes responding better to chemotherapy than non‐TNBC.^11^, ^30^ The lack of target receptors (e.g., ER/PR or HER‐2), means that TNBC patients do not benefit from endocrine or targeted therapies. Therefore, surgery and chemotherapy (alone or in combination) are the modalities available for TNBC. Anthracyclines (A) and taxanes (T) are the mainstay of chemotherapy regimens with the recent addition of platinum‐based agents.

### 3.1 Neoadjuvant therapeutic agents

**FIGURE 2:**
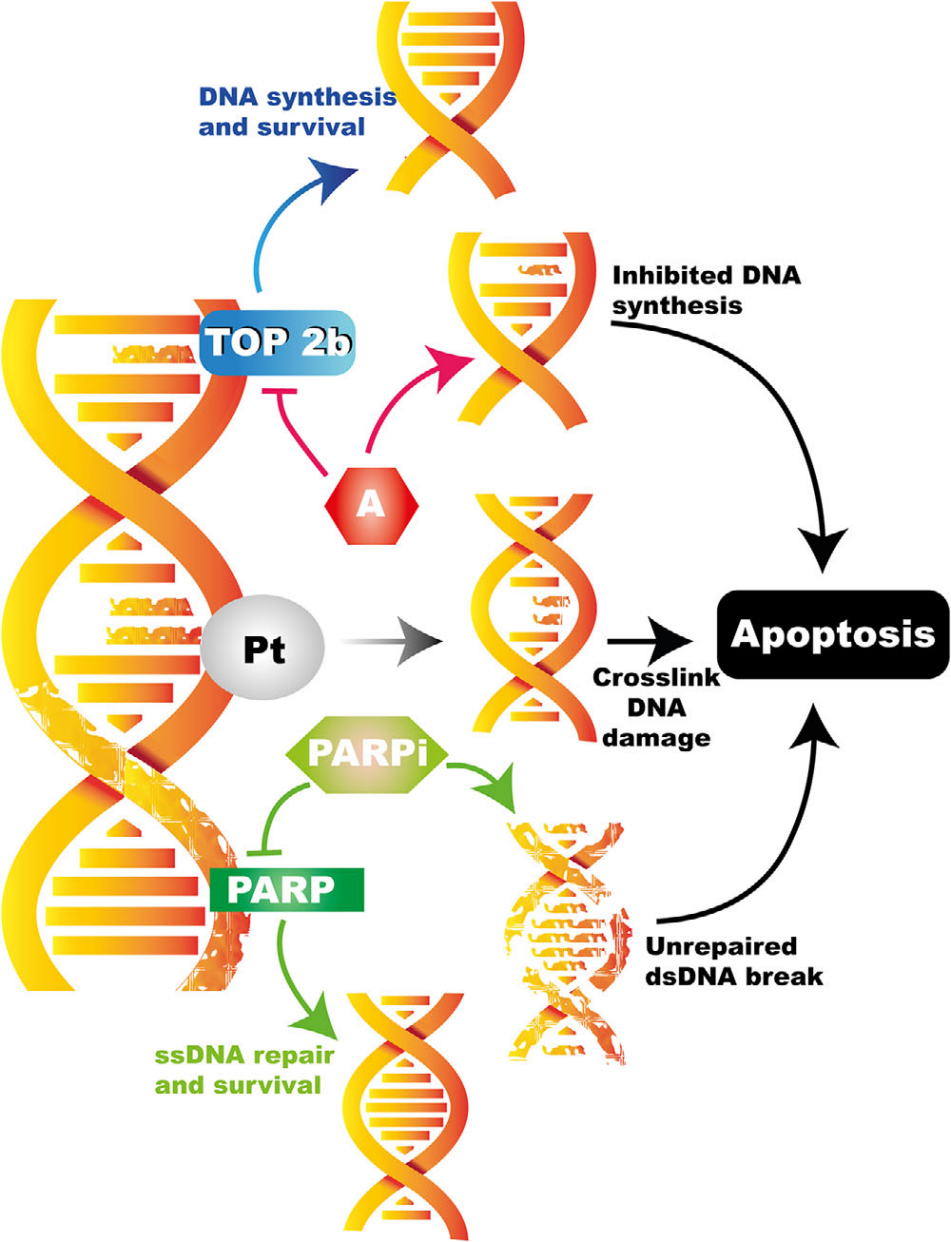
Current TNBC therapeutics aim to disrupt DNA structure thereby leading to DNA damage and consequent cell death. Chemotherapeutic agents such as anthracyclines (A) inhibit molecules required for DNA synthesis. Platinum‐based compounds (Pt) target DNA cross‐linking resulting in cell apoptosis. PARP inhibitors prevent the repair of single strand DNA (ssDNA) damage. The accumulation of ssDNA damage results in unrepairable double strand DNA (dsDNA) breaks leading to cell death

Neoadjuvant chemotherapy is commonly used to treat patients with early TNBC, aiming to target DNA repair and cell proliferation mechanisms (Figure 2). A study looking at the relationship between anthracycline‐based neoadjuvant chemotherapy (doxorubicin plus cyclophosphamide, AC) and long‐term endpoints in different subtypes of BC showed that TNBC patients (*n *= 34) demonstrated the highest clinical response rates compared to HER‐2 (*n *= 11) and ER‐positive (*n *= 62) BCs (85% vs 70% vs 47%, respectively).^31^ Moreover, pCR rates, defined as the absence of residual invasive tumor in breast and regional nodes at the time of surgery, were significantly higher in TNBC (27%) than ER‐positive patients (7%). A larger study described similar findings where higher pCR rates are observed in TNBC (*n *= 255) than in non‐TNBC (*n *= 893) in response to neoadjuvant AC chemotherapy (30% vs 6.7%, respectively).^30^ However, in both of the above studies, TNBC OS and distant‐free survival were significantly lower than for non‐TNBC.^31^, ^32^ Following these findings, the NSABP B‐27 trial evaluated long‐term outcomes in response to AC therapy with the addition of docetaxel (T).^33^ Here, 2411 women were assigned into one of three arms; the first arm received AC followed by surgery, the second received AC followed by T and then surgery, and the third arm received AC followed by surgery then T. The addition of T to AC preoperatively resulted in an increase in pCR rates compared to AC alone in TNBC patients (22.8% vs 13.6%, respectively); however, an updated analysis of the study showed that the addition of T did not improve DFS or OS in these patients.^34^

Platinum‐based compounds (such as cisplatin and carboplatin) have also been considered as treatment in TNBC due to their DNA cross‐linking properties (Figure 2), consequently resulting in tumor cell apoptosis.^35^ The Alliance phase 2 trial (Cancer and Leukemia Group B 40603) studied pCR rates in 443 stage 2–3 TNBC patients in response to carboplatin treatment with addition of paclitaxel (followed by AC).^36^ pCR rates were significantly higher in patients treated with carboplatin, compared to those that received paclitaxel and doxorubicin alone (60% vs 44%; *p* = 0.0018).^36^ Similar results were observed in the parallel GeparSixto trial, also in stage 2–3 TNBC patients receiving paclitaxel and doxorubicin neoadjuvant chemotherapy with the addition of weekly carboplatin treatment.^37^ Here, carboplatin‐treated patients demonstrated 53.2% pCR (compared to 36.9%, *p* = 0.005) without platinum‐based therapy, but significantly more toxic effects were observed in the carboplatin group than the no‐carboplatin group (neutropenia: 65% vs 27%, anemia 15% vs 1%, diarrhea 17% vs 11%).^37^ However, DFS and OS were not assessed in these studies; thus, the hypothesis that OS benefit can be predicted by increased rates of pCR, as proposed by Cortazar et al.,^38^ remains controversial.

### 3.2 Addition of immunotherapy

Approximately 20% of TNBC cases are highly enriched in immune cell markers, and these are classified as immunomodulatory. Tumors that possess more than 50% tumor‐infiltrating lymphocytes (TILs) are associated with better prognosis with improved OS, increased metastasis‐free survival, and decreased distant recurrence.^39^ The immune‐checkpoint receptor, PD1, and its ligand, PD‐L1, are correlated with high levels of TILs in the BC microenvironment and is one of the most common subtypes in TNBC.^40^, ^41^ The overexpressed PD‐L1 (on tumor cells) binds to the PD1 receptor of activated T‐cells, thereby inhibiting their cytotoxic activities on the tumor cell. Thus, targeting the PD1–PD‐L1 axis has become an attractive approach in TNBC because PD‐L1 is expressed in 20% of all TNBC cases.^42^ PD1 inhibitors such as pembrolizumab have proven to be effective in the treatment of lung, melanoma, and bladder cancers.^43^, ^44^, ^45^ In the assessment of safety and antitumor activity of pembrolizumab in TNBC, a phase‐1b (KEYNOTE‐012) trial enrolled 111 TNBC patients, 58.6% of which expressed PD‐L1‐positive tumors.^46^ From these, mild toxicities were noted such as arthralgia, fatigue, and nausea with only five patients exhibiting grade ≥ 3 toxicity and the overall response rate was 18.5% (ClinicalTrials.gov identifier: NCT02447003). Currently, an ongoing open‐label, adaptively randomized phase‐2 trial will assess pembrolizumab plus neoadjuvant chemotherapy in stage 2/3 BC patients (ClinicalTrials.gov identifier: NCT01042379). The latest results from this study (obtained in 2017) demonstrated pCR rates of 44% versus 17% (HER2‐negative), 30% versus 13% (HR‐positive), and 60% versus 22% (TNBC) for pembrolizumab versus control, respectively.^47^ In a similar phase‐3 trial (IMpassion031), atezolizumab plus chemotherapy was assessed in the neoadjuvant setting compared to placebo plus chemotherapy (ClinicalTrials.gov identifier: NCT03197935). It has been recently reported that pCR rates improved with atezolizumab plus chemotherapy compared to the placebo plus chemotherapy arm (57.6% vs 41.1%, respectively), particularly in PD‐L1‐positive patients with pCR rates reaching 68.8%.^48^ Further follow‐up of patients on these studies will establish whether the improved pCR translates into increased DFS or OS.

### 3.3 Adjuvant chemotherapy

Optimizing early‐stage chemotherapy in TNBC is imperative for reducing the risk of recurrence, distant metastases, and eventual death. Recent guidelines set by the European Society for Medical Oncology (ESMO) do not recommend further adjuvant therapy for patients with TNBC if residual disease is present after completion of neoadjuvant chemotherapy.^49^ Despite this, a number of trials have shown that statistically significant improvements were observed in DFS and OS when patients also received adjuvant chemotherapy.^50^, ^51^ For example, a randomized study assessing the effects of capecitabine following neoadjuvant chemotherapy in 910 patients with HER2‐negative residual invasive BC described longer DFS and OS compared to a noncapecitabine‐treated control group (74.1% vs 67.6% and 89.2% vs 83.5%, respectively).^51^ For the patients with TNBC (32.2% of the study population), DFS was 69.8% in the capecitabine group and 56.1% in the control group and OS was also significantly improved (78% vs 70.3%). Adjuvant immunotherapy has also been considered postneoadjuvant chemotherapy in TNBC. The current multicenter phase 2 c‐TRAK‐TN trial utilizes circulating tumor DNA (ctDNA) screening to detect residual disease following primary treatment for TNBC (ClinicalTrials.gov identifier: NCT03145961). Originating from tumor cells, ctDNA are extracellular DNA molecules found in the plasma or serum of cancer patients. ctDNA screening allows for early cancer detection and is able to determine the tissue of origin, prognosis, and detection of minimal residual disease.^52^ In the instance of a positive ctDNA result, patients will be randomized into a pembrolizumab treatment arm or an observation arm. Although focused on the utility of ctDNA in monitoring disease progression, this study also includes descriptive differences in time between ctDNA detection and disease recurrence, and DFS, between patients in the pembrolizumab and the observation groups, among the outcome measures.

Conversely, a number of studies have reported no differences in prognosis or survival rates between TNBC patients who received only adjuvant anthracycline‐ and nonanthracycline‐based chemotherapy.^53^, ^54^, ^55^, ^56^ However, dose dense adjuvant anthracycline‐based chemotherapy has demonstrated good survival advantages. Increasing the dose intensity of chemotherapy by shortening the intervals between cycles or by administering individual drug sequentially at full dose may improve efficacy. A recent meta‐analysis conducted by the Early Breast Cancer Trialists’ Collaborative Group (EBCTCG) studied the benefits of dose‐intense chemotherapy comparing 2‐weekly *versus* 3‐weekly schedules reported from 26 trials.^57^ Patients receiving dose‐intense chemotherapy exhibited lower recurrences compared to standard‐schedule chemotherapy (10‐year recurrence risk 28.0% vs 31.4%). Ten‐year BC mortality and death without recurrence were also lower in response to dose‐intense chemotherapy than standard schedule (18.9% vs 21.3% and 4.1% vs 4.6%, respectively). Similar reductions in recurrence were observed in 2‐weekly chemotherapy compared to the same treatment given 3‐weekly (10‐year risk; 24% vs 28.3%). This was also observed when anthracycline plus taxanes chemotherapy was administered sequentially as opposed to concurrently (28.1 vs 31.3%). These differences may arise due to heterogeneity of TNBC disease. The success of anthracycline‐based therapies depends on the TNBC subtype and/or the underlying genetic factor (for example, BRCA1 mutations), as it is even suggested that BRCA1‐associated TNBC may be less sensitive to anthracycline‐based therapies compared to sporadic TNBC.^58^ *BRCA1/2* are tumor suppressor genes that play important roles in DNA damage repair, cell cycle checkpoint control, apoptosis, and transcriptional regulation.^59^ Thus, mutations in *BRCA1/2* induce defects in the DNA damage repair processes that are associated with the risk of development of BC.^60^ The OlympiA phase‐3 trial assessed olaparib, a poly(adenosine diphosphate‐ribose) polymerase inhibitor, in patients with TNBC (and ER+HER2‐) germline BRCA1/2 mutations (ClinicalTrials.gov identifier: NCT02032823). In this study, 1836 participants were enrolled and randomized (1:1) into an olaparib arm or a placebo arm. Patients underwent 12 months of treatment and recent interim results have shown that olaparib resulted in significant improvements in 3 year invasive DFS (85.9% vs 77.1% in the placebo group), 3 year distant DFS (87.5% vs 80.4% in the placebo group), as well as fewer deaths (59 compared to 86 in the placebo group).^61^ However contradictory results are still being reported in a number of studies relating to prognosis of neo‐ and adjuvant chemotherapy^62^, ^63^, ^64^; thus, the search for alternative therapies has become an imperative avenue for exploration.

### 3.4 Treatment of metastatic TNBC

Once metastases develop, biopsy assessment is conducted when clinically achievable, to confirm hormone receptor and HER‐2 status, as 8% of tumors that were ER negative convert to ER positive at the metastatic site.^65^, ^66^ Consequently, modification of the therapeutic approach is necessary upon reevaluation of metastatic disease.^67^ Cytotoxic chemotherapy is currently the backbone of first‐line treatment options for mTNBC and is aimed to prolong survival, palliate symptoms, and delay disease progression. Current guidelines recommend that systemic chemotherapy should be individualized based on tumor burden, rate of disease progression, previous chemotherapy treatments, and patient preferences.^68^ More recently, the addition of platinum‐based agents to first‐line chemotherapy has been suggested as a more effective approach in mTNBC. In a retrospective cohort study (*n *= 379), patients treated with platinum‐based chemotherapy demonstrated longer PFS compared to nonplatinum‐based chemotherapy (7.8 vs 4.9 months).^69^ Additionally, the phase‐3 Triple Negative Breast Cancer Trial (TNT) compared carboplatin with docetaxel in 400 patients with either mTNBC or with known *BRCA1/2* mutations.^70^ A 2014 snapshot analysis showed that there were no statistically significant differences in PFS (carboplatin 3.1 months vs docetaxel 4.5 months) and OS (carboplatin 12.3 months vs docetaxel 12.4 months) in the TNBC group. On the other hand, PFS and objective response rates were improved in response to carboplatin in the *BRCA1/2* carriers (6.8 months vs 3.1 months and 68% vs 33%, respectively).^71^ With these data, a platinum‐based chemotherapy regimen has been recommended by the European Society of Medical Oncology for patients with *BRCA‐*associated TNBC, if not previously administered.^49^

The phase‐3 study, KEYNOTE‐355, with pembrolizumab in mTNBC or inoperable TNBC is being undertaken in two parts (ClinicalTrials.gov identifier: NCT02819518); the first aims to assess the safety of pembrolizumab in combination with chemotherapy on tumors that have not been previously treated with chemotherapy. The second part of the study will compare the safety and efficacy of pembrolizumab plus chemotherapy to placebo plus chemotherapy in the aim that pembrolizumab, in combination with chemotherapy, will prolong progression free survival (PFS) and OS. In parallel, the KEYNOTE‐522 study will assess the safety and efficacy of pembrolizumab plus chemotherapy as neoadjuvant therapy (ClinicalTrials.gov identifier: NCT03036488). Here, after screening and randomization, patients with locally advanced TNBC will receive pembrolizumab plus chemotherapy or placebo plus chemotherapy for 24 weeks (eight cycles). Each patient will then undergo definitive surgery in which adjuvant treatment of pembrolizumab or placebo will be administered for a further 27 weeks (nine cycles). Patients will be monitored for safety, survival, and disease recurrence. A phase‐3 trial (Impassion130) assessed atezolizumab (anti – PD‐L1 antibody) plus nab‐paclitaxel *versus* placebo plus nab‐paclitaxel in mTNBC.^72^ Here, 451 patients were assigned to each group in which PFS and OS were improved in the atezolizumab plus nab‐paclitaxel treatment arm (PFS: 7.2 months vs 5.5 months, OS: 21.3 months vs 17.6 months, respectively). In contrast, the IMpassion131 (phase‐3) trial reported no differences in the treatment arms when atezolizumab and paclitaxel were combined compared to placebo plus paclitaxel (ClinicalTrials.gov identifier: NCT03125902). Nab‐paclitaxel and paclitaxel are drugs from the same class though differ in delivery method. Nab‐paclitaxel is albumin‐bound, whereas paclitaxel is delivered in a solvent and requires pretreatment with steroids. Although yet not clear, this difference maybe a contributing factor to the conflicting results. From these results, all TNBC patients are now PD‐L1 checked at diagnosis of metastatic disease and, following the approval by the FDA, atezolizumab plus nab‐paclitaxel is used as first‐line therapy in PD‐L1‐positive TNBC.

In addition to targeting the PD‐L1 axis, DNA‐targeting molecules such as PARP inhibitors have shown efficacy in mTNBC. The *BRCA1* and *BRCA2* tumor suppressor genes code for proteins involved in the DNA damage‐sensing process and double‐stranded DNA break repair mechanisms. *BRCA1* mutations are present in 50–87% of TNBC patients.^73^, ^74^, ^75^ In addition to *BRCA1*, the PARP1 and PARP2 enzymes are also activated by DNA single‐strand breaks that subsequently facilitate DNA repair, essential processes for cancer cell survival (Figure 2). In the absence of PARP, the accumulation of single‐strand breaks results in cytotoxic double‐strand breaks that would normally be rectified by *BRCA*.^76^ However, *BRCA1*‐mutated BC lacks this mechanism; thus, inhibiting PARP poses as an attractive therapeutic approach in *BRCA1*‐associated TNBC. Studies using in vitro and in vivo models of *BRCA1/2* TNBC have shown sensitivity to PARP inhibitors, demonstrating significant tumor regression and longer DFS and OS in mice.^77^, ^78^, ^79^, ^80^ The recent olympiAD trial (ClinicalTrials.gov identifier NCT02000622) studied the PARP inhibitor olaparib in 302 mBC patients with known *BRCA* mutations.^81^ Patients were randomized to single‐agent chemotherapy (*n* = 97) or olaparib (*n* = 205); 49.8% of patients in the olaparib group and 49.5% of the chemotherapy group exhibited TNBC disease. Response rates and PFS were significantly higher in the olaparib compared to chemotherapy group (59.9% vs 28.8% and 7 vs 4.2 months, respectively), and hence, olaparib was approved for the treatment of metastatic HER‐2‐negative BC patients with *BRCA* mutations by the FDA in January 2018. A trial of Talazoparib (EMBRACA; ClinicalTrial.gov identifier: NCT01945775), another PARP inhibitor, also resulted in significantly improved PFS and ORR (objective response rate) rates compared to standard therapy (DFS; 8.6 vs 5.6 months, ORR; 62.6% vs 27.2, respectively).^82^ Talozoparib is now also FDA approved in patients pretreated with chemotherapy with mTNBC. In addition to *BRCA1/*2 mutations, a number of (mutated) genes have been identified as biomarkers in TNBC. For example, an inactivating mutation of *TP53* that is also involved in DNA damage repair and genome integrity is associated with poor prognosis due to poor responses to chemotherapy.^83^ Other biomarkers such as *PTEN*, *PIK3CA*, and *EGFR* have been described in playing roles in TNBC and reviewed in detail by Sporikova et al.^84^ More recently, a phase‐3 trial (ASCENT) assessed the trophoblast cell‐surface antigen 2 (Trop‐2) as a potential biomarker for the treatment of mTNBC using the Trop‐2 directed antibody‐drug conjugate sacituzumab govitecan (SG).^85^ It is understood that elevated levels of Trop‐2 are associated with tumor growth in TNBC, poor prognoses, and decreased survival.^86^, ^87^ Biopsy samples of patients with mTNBC were therefore taken to determine Trop‐2 expression levels and were randomized to receive SG (10 mg/kg) or TPC (apecitabine, eribulin, vinorelbine, or gemcitabine). It was found that patients with high and medium expressions of Trop‐2 benefited from SG treatment compared to those with low expression. For example, PFS in SG‐treated and TPC‐treated patients was 6.9 versus 2.5 (high Trop‐2) 5.6 versus 2.2 (medium Trop‐2) and 2.7 versus 1.6 (low Trop‐2).^85^ This was also the case for OS (14.2, 14.9, and 9.3 months versus 6.9, 6.9, and 7.6 months) and ORR (44%, 38%, and 22% versus 1%, 11%, and 6%) for SG‐ and TPC‐treated patients with high, medium, and low Trop‐2 expression levels, respectively. Interestingly, it was also found that BRCA1/2 status did not impact the effects of SG as PFS and OS were improved in BRCA1/2‐positive (PFS: 4.6 vs 2.5 months, OS: 15.6 vs 4.4 months SG and TPC, respectively) and BRCA1/2‐negative patients (PFS: 4.9 vs 1.6 months, OS: 10.9 vs 7 months SG and TPC, respectively). This study illustrates the importance of identifying suitable biomarkers, in this case to target DNA repair factors, which allow for successful targeted therapies.

As well as targeting PARP directly, findings from a recent study suggest that targeting PARP‐associated molecules could also prove to be interesting therapeutic approaches. For example, the transcription factor KLF4 (involved in a number of cellular processes such as cell cycle control and genome stability) undergoes PARP‐mediated parylation.^88^ More specifically, parylation of KLF4 on the YYR motif by PARP is essential for the survival of cancer cells in which both proteins have been found to be overexpressed in TNBC cell lines.^88^ Disruption of the KLF4‐PARP axis by knocking out KLF4 (KLF4^−/−^) resulted in a dysfunctional DNA damage response in TNBC cell lines. Furthermore, when exposed to the PARP inhibitor, olaparib, KLF4^−/−^ cells significantly enhanced olaparib‐induced cell death in BRCA1‐proficient TNBC cell lines. In contrast, BRCA1‐defient TNBC cell lines did not respond to olaparib regardless of KLF4 status. The sensitizing efficacy for olaparib in BRCA1‐proficeint tumors was also demonstrated in vivo where KLF4^−/‐^ xenograft tumor growth was significantly perturbed compared to control KLF4^−/−^ tumors. Analysis of these tumors showed that KLF4^−/‐^ resulted in decreased proliferation and increased cell death in response to olaparib treatment. With such findings from preclinical studies, and clinical outcomes that have provided patients with small successes, the identification of alternative therapeutic targets in TNBC remains a high clinical priority while taking major challenges such as tumor heterogeneity and biomarkers into consideration.^89^

## 4 CYCLIN‐DEPENDENT KINASE INHIBITORS IN METASTATIC TNBC

### 4.1 Preclinical studies of CDK4/6 inhibitors in TNBC

**FIGURE 3:**
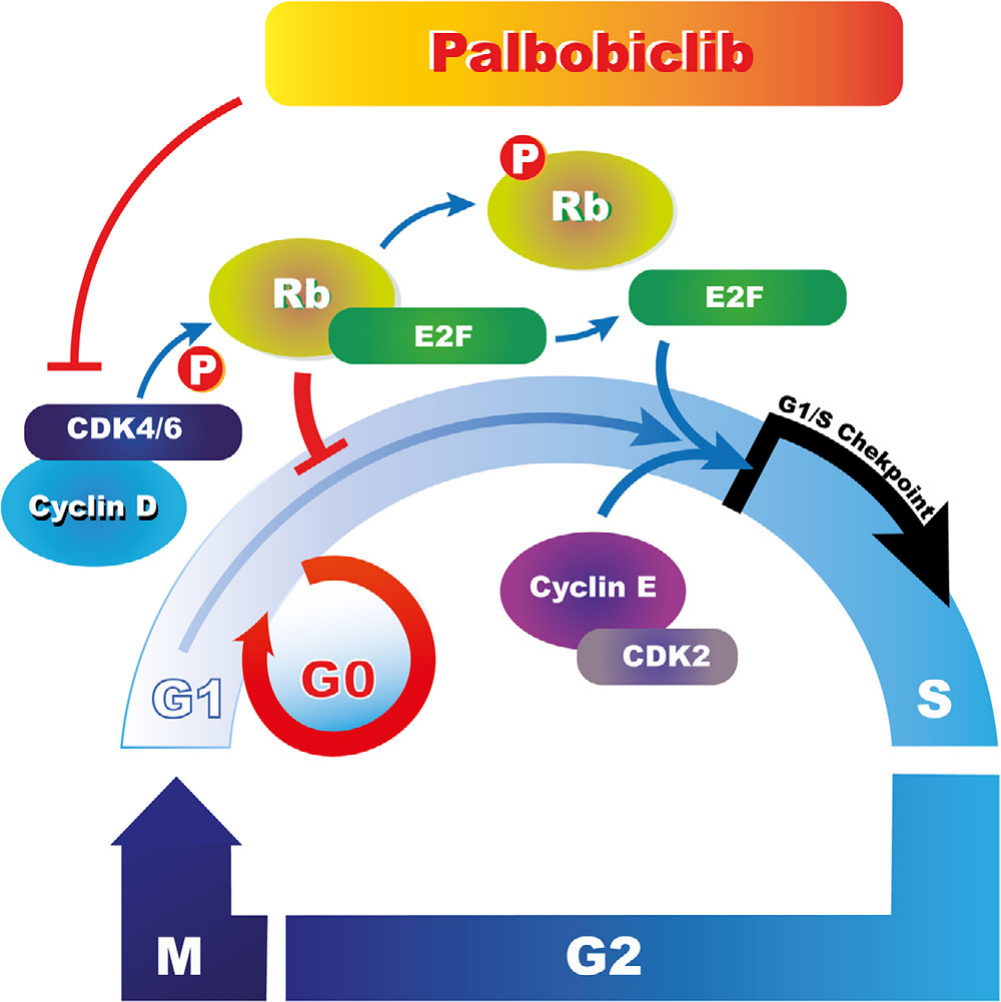
CDK 4/6 mediated cell cycle progression. Under normal conditions, the phosphorylation of the retinoblastoma protein (Rb) by the CDK 4/6—cyclin D complex results in its dissociation from E2F thereby allowing for the transcription of genes for cell cycle progression. CDK4/6 inhibitors such as Palbociclib aim to prevent the progression of the cell cycle and maintain cell cycle arrest

Cyclin‐dependent kinase (CDK) 4/6 is a key regulator of the transition from the G1 phase of the cell cycle and initiates cell cycle progression. In this pathway, cyclin D1 forms an activating complex with CDK 4 and CDK6 that go on to phosphorylate the retinoblastoma protein (Rb). Once phosphorylated, phosphor‐Rb (pRb) binds to the E2F transcription factors that subsequently regulate the expression of a series of genes that initiate progression through the cell cycle (Figure 3).^90^ In cancer, this pathway is dysregulated, resulting in aberrant cell proliferation.^91^ Almost 50% of BC patients exhibit cyclin D1 overexpression, and this, in turn, results in the phosphorylation of Rb and progression of the cell cycle.^92^ This has led to the development of CDK4/6 inhibitors that have proved successful, alone and in combination with endocrine therapy, in ER‐positive BC. Importantly, not only were direct anticancer effects observed, but sensitization of endocrine therapy‐resistant BCs was induced.^4^, ^93^

In TNBC, Rb dysfunction occurs in approximately 30% of cases.^94^ Additionally, from 180 TNBC patient samples, 51% were found to be Rb positive, thus representing a relevant target for therapy.^95^ Early in vitro studies aimed to investigate of the effects of CDK4/6 inhibition to identify potential targetable pathways in TNBC.^93^, ^96^ Using the CDK4/6 inhibitor palbociclib, Finn et al. showed that growth of a panel of TNBC cell lines was inhibited, although less sensitive to CDK4/6 inhibition than ER‐positive cell lines.^93^ A more detailed analysis, using a panel of 12 TNBC cell lines, showed that LAR TNBC cells demonstrated higher sensitivity compared to basal‐like and MES lines that were found to be resistant to the CDK4/6 inhibitors palbociclib and ribociclib.^96^ This was also the case for tumors grown in vivo; LAR xenograft tumors were significantly reduced in size when treated with palbociclib compared to the vehicle‐treated group.^96^ This limited evidence suggests that particular TNBC subgroups may be sensitive to CDK4/6 inhibition and further preclinical studies are needed to establish how these can be identified.

The mechanisms responsible for CDK4/6 resistance are poorly understood; however, the loss of Rb (observed in 7–20% of TNBC) and overexpression of cyclin E have been shown to confer resistance to CDK4/6 inhibitors.^97^, ^98^, ^99^ Asghar et al. describe that when exiting mitosis, palbociclib‐sensitive TNBC cells exhibit lower levels of CDK2 compared to palbociclib resistant cells.^96^ The cyclin E–CDK2 axis also plays an important role in the regulation of the cell cycle^100^; therefore, the Rb and CDK2 status may act as biomarkers for CDK4/6‐targeted inhibition. In a more recent study, investigators assessed palbociclib in combination with the chemotherapeutic agent paclitaxel in vitro.^101^ When MDA‐MB‐231 cells were exposed to a combination of palbociclib and paclitaxel, an antagonistic effect was observed, where the inhibition of cell proliferation caused by palbociclib impeded the cytotoxic effects of paclitaxel. However, sequential treatment with palbociclib followed by paclitaxel resulted in an additive inhibitory effect of cell proliferation.^101^ Cell death was also significantly higher following exposure to palbociclib then paclitaxel, compared to that caused by palbociclib or paclitaxel alone. These data demonstrate the importance of palbociclib being administered first when given in sequence with antiproliferative drugs, as this allows tumor cells to reenter the cell cycle synchronously once palbociclib is removed and thus sensitizing cells to the effects of chemotherapeutic agents.

Outside the CDK4/6 axis, it has also been shown that palbociclib reduces glucose metabolism by downregulating the GLUT‐1 glucose transporter in the TNBC MDA‐MB‐231 cell line.^102^ Glucose uptake and consumption were further inhibited when cells underwent sequential exposure to palbociclib followed by paclitaxel.^101^ Enzalutamide, an AR antagonist that is commonly used in the treatment of prostate cancer, has also shown antitumor effects in TNBC because 30% of TNBC patients demonstrate AR‐positive disease.^103^, ^104^, ^105^ The combination of palbociclib and enzalutamide resulted in an enhanced cytostatic effect compared to palbociclib and enzalutamide alone, with no cell death observed in the TNBC cell lines.^106^ In the metastatic setting, an in vivo study investigating the effects of palbociclib on distal site invasion found a significant decrease in liver (12% vs 75%) and lung (25% vs 75%) metastases (compared to saline treated mice) in TNBC xenograft models.^107^ Treatment with palbociclib, initiated after the resection of the primary tumor, inhibited lung colonization resulting in a significantly lower number of lung nodules compared to the saline treated control group. In the mechanism of invasion and EMT, SNAIL1 is a key regulator of this process by repressing the expression of *CDH1* (the gene encoding E‐cadherin) and activating the expression of invasion‐associated genes.^108^, ^109^ Upon treatment with palbociclib, a decrease in SNAIL1 protein stability and increased ubiquitination (marking proteins for degradation) was observed in TNBC cells. It was also shown that palbociclib did not directly interact with SNAIL1, but instead acted through the phosphorylation of deubiquitinating enzyme 3 (DUB3), which, in turn, downregulates SNAIL1,^107^ suggesting a new target for palbociclib. Separately, a recent study investigated the effects of palbociclib treatment preceding cisplatin chemotherapy.^110^ In the MDA‐MB‐231 and MDA‐MB‐468 cell lines, palbociclib alone resulted in cell cycle arrest as expected, but when cells were treated in combination with cisplatin, cell apoptosis was unaffected. This is due to the inability of the chemotherapeutic agent to act on already arrested cells. However, when cells were treated with palbociclib for 48 h followed by its removal for 48 h, then treated with cisplatin for 48 h, significantly increased apoptosis was observed compared to monotherapy. Additionally, sequential treatment resulted in increased DNA damage and lower cell viability compared to single drug treatments. The MDA‐MB‐231 xenograft model was used to study the effects of sequential treatment in vivo. Mice were treated with vehicle (PBS), palbociclib only, cisplatin only, or palbociclib followed by cisplatin 48 h later. Palbociclib alone did not affect tumor volume and weight, whereas cisplatin alone caused a significant decrease compared to vehicle control.^110^ Further inhibition in tumor growth was observed in the sequential treatment group, with lower tumor Ki‐67 expression than that seen in single treatments and vehicle controls. Thus, pretreatment with palbociclib sensitizes cells to cisplatin and further increases its antitumor effect. Taken together, these preclinical studies suggest that targeting the CDK4/6 signaling pathway, sequentially with current chemotherapy agents, provides an alternative therapeutic approach in TNBC. In addition to providing alternative approaches, the safety of CDK4/6 inhibitors in combination with current therapies must be vigorously assessed. CDK4/6 inhibitor use in ER‐positive BC has proved to be a safe option when combined with hormone therapies^4^; however, little data of the safety of CDK4/6 inhibitors exist and are discussed below

### 4.2 CDK4/6 inhibitors in treatment of TNBC—Clinical studies

In ER‐positive BC, CDK4/6 inhibitors have made major advancements in improving DFS and OS, particularly in combination with endocrine therapies as recently reviewed.^4^ The success of CDK4/6 inhibitors such as abemaciclib (MONARCH studies), ribociclib (MONALEESA studies), as well as palbociclib (PALOMA studies), in combination with endocrine therapy, has been demonstrated in a number of trials in ER‐positive BC.^111^, ^112^, ^113^, ^114^, ^115^, ^116^ Recently, the safety of ribociclib plus tamoxifen (or letrozole) was assessed in 672 ER‐positive, HER‐2 negative BC patients in the MONALEESA‐7 trial.^117^ A total of 335 patients were assigned to the ribociclib group and 337 to the placebo group. Here, median PFS was significantly higher in the ribociclib group compared to the placebo group (23.8 months vs 13 months, respectively). Follow‐up analysis found that 24.8% of deaths occurred in the ribociclib group compared to 32.3% in the placebo group; thus, OS was significantly higher in the ribociclib group than the placebo group (42 months vs 46 months, respectively).^118^ In addition to these findings, a study found that ribociclib plus letrozole resulted in greater cost‐savings than other CDK4/6 inhibitor‐letrozole combination making it a cost‐effective treatment for ER‐positive BC.^119^

In contrast, little data exist for CDK4/6 inhibitors in clinical trials in TNBC (Table 1), despite preclinical studies demonstrating that TNBCs express CDK4/6 and their growth is inhibited by CDK4/6 inhibitors, as described above. In 2015, a phase‐2 clinical trial assessing the safety of palbociclib, where 11% of the 37 patients enrolled had TNBC disease.^120^ Unfortunately, no further TNBC patients were recruited due to the rapid disease progression observed in all four TNBC patients. These patients also showed a significantly lower PFS compared to ER‐positive patients (1.5 months vs 4.5 months) in response to palbociclib treatment. At the same time, a nonrandomized phase‐1/2 open‐label, single‐arm trial studied the effects of palbociclib and bicalutamide (anti‐AR agent) (ClinicalTrials.gov identifier: NCT02605486). Thus far, the combination of palbociclib and bicalutamide has proven to be safe and well tolerated by AR‐positive TNBC patients, with no dose‐limiting toxicities recorded or grade 4 or 5 adverse events.^121^ The study aims to establish the recommended phase‐2 dose of combination as well as overall response rates and 1‐year PFS, though as of yet no clinical data have been recorded. Additionally, the phase‐1 PAveMenT trial (still recruiting at the time of writing) will test the safety of using palbociclib and avelumab in combination, as it is hypothesized that this may be an effective approach compared to sequential treatment in AR‐positive TNBC patients (ClinicalTrials.gov identifier: NCT04360941). Similarly to palbociclib, ribociclib has been investigated in an open‐label phase‐1/2 trial in combination with bicalutamide in AR‐positive TNBC disease to assess toxicity and clinical benefit rate (ClinicalTrials.gov identifier: NCT03090165—though patient recruitment is yet to commence at the time of writing this review). A third CDK4/6 inhibitor, abemaciclib, has also been investigated in TNBC. A phase‐1 trial assessing the efficacy and safety of abemaciclib reported nine (out of 225) patients had TNBC demonstrated only 1.1 months of median PFS and a clinical benefit rate of 11%.^122^ Although results from this trial were more promising for ER‐positive patients, other trials have been established to identify better strategies for TNBC. One such approach is the single‐arm phase‐2 trial that is currently recruiting an estimated 37 metastatic Rb‐positive TNBC patients (invasive tumor has > 50% of Rb‐positive cells) that are to receive abemaciclib as a single agent (ClinicalTrials.gov identifier: NCT03130439). Results from these trials will help pave the way for CDK4/6 inhibitors to be incorporated into existing therapeutic strategies for TNBC.

**TABLE 1:** Clinical trials with CDK4/6 inhibitors in TNBC obtained from ClinicalTrials.gov

Trial ID	Phase	Arms	Treatment plan	Disease	Primary outcome	Results	Status	REF
	Abemaciclib							
NCT03130439	II	Single arm: Abemaciclib	150 mg twice daily for 28 days	Rb‐positive metastatic TNBC	ORR	Awaiting	Recruiting	
Patnaik et al.	I	Single arm: Abemaciclib	once daily: 50 mg (*n* = 4), 100 mg (*n* = 3), 150 mg (*n* = 3), or 225 mg (*n* = 3) Twive daily: 75 mg (*n* = 3), 100 mg (*n* = 4), 150 mg (*n* = 3), 200 mg (*n* = 7), and 275 mg (*n* = 3)	TNBC	Safety and tolerability	Median PFS 1.1 months (vs HR+ 8.8 months)		122
	Palbociclib							
NCT02605486	I/II	Palbociclib + Bicalutamide	Phase I: to be determined Phase II: bicalutamide orally once daily. Palbociclib will be given orally daily for 3 weeks on followed by 1 week off at the doses determined in phase I	AR‐positive metastatic TNBC	Phase II dose Phase II PFS	Awaiting	Active: not recruiting	
DeMichele et al.	II	Single arm: Palbociclib	125 mg for 3 weeks on followed by 1 week off	Rb‐positive metastatic TNBC		Median PFS 1.5 months (vs HR+/Her2−: 3.8 months vs HR+/Her2+: 5.1 months)		120
NCT04360941	I	Palbociclib + Avelumab	Part A: palbociclib dose escalation + fixed dose of avelumab (n = 18) Part B: MTD and schedule determined by plan A.	AR‐positive metastatic TNBC		MTD ORR	Recruiting	
	Ribociclib							
NCT03090165	I/II	Ribociclib + Bicalutamide	Phase I: cohort 1: bicalutamide 150 mg daily on days 1–28 + ribociclib 400mg daily on days 1–21 of a 28 day cycle cohort 2: bicalutamide 150 mg daily on days 1–28 + ribociclib 400 mg daily on days 1–28 of a 28 day cycle cohort 3: bicalutamide 150 mg daily on days 1–28 + ribociclib 600 mg daily on days 1–21 of a 28 day cycle Phase II: maximum safe dose of ribociclib in combination with bicalutamide (*n* = 25)	Rb‐positive metastatic TNBC	Phase I MTD Phase II CBR	Awaiting	Recruiting	

Abbreviations: ORR, objective response rate; PFS, progression free survival; CBR, clinical benefit rate; MTD, maximum tolerated dose.

## 5 ALTERNATIVE PATHWAY INHIBITORS IN COMBINATION WITH CDK4/6 INHIBITION

### 5.1 mTOR/PI3K inhibitors

Alternative pathways that also regulate cell proliferation, survival, and growth, such as the mTOR/PI3K and PARP pathways, have been shown to be deregulated in BC.^123^, ^124^, ^125^ These pathways have therefore been targeted in ER‐positive BC through agents such as the PIK3 inhibitor, BYL719, in combination with ribociclib and letrozole (*AI*) (ClinicalTrials.gov : NCT 01872260), or the mTOR inhibitor, everolimus plus ribociclib and exemestane (*AI*) (ClinicalTrials.gov : NCT 01857193).^4^, ^126^ In TNBC disease, the combination of ribociclib and alpelisib (a PI3K inhibitor) demonstrated significantly increased apoptosis and cell cycle arrest in Rb‐positive TNBC cell lines in vitro.^127^ In addition, in vivo xenograft models of TNBC demonstrated improved disease control in response to combined CDK4/6 and PI3K inhibition. Combined inhibition of CDK4/6, PI3K, and immune checkpoint pathways resulted in complete regression of established TNBC tumors.^127^ The mTOR and PI3K pathway are closely linked and often considered as a single pathway. The mTOR kinase inhibitor, MLN0128, has been tested in combination with palbociclib in TNBC cell lines.^128^ In this study, cell proliferation was significantly inhibited through cell cycle arrest and western blot analysis showed inhibition of the CDK4/6‐Rb and mTOR pathways in response to combination treatment.^128^ Moreover, in TNBC patient‐derived xenograft models, palbociclib or MLN0128 monotherapy, resulted in significantly inhibited tumor growth. However, when used in combination, further significant tumor growth inhibition was observed compared to monotherapy or the saline control group. As previously mentioned, palbociclib has inhibitory effects on glucose metabolism outside the CDK4/6‐Rb axis. When palbociclib is combined with alpelisib or BEZ235 (a dual PI3K and mTORC1‐2 inhibitor), an additive inhibitory effect is observed on cell proliferation in the TNBC MDA‐MB‐231 and MDA‐MB‐468 cell lines.^102^ Moreover, preincubation with palbociclib for 24 h, followed by simultaneous PI3K inhibition, resulted in a synergistic inhibition of cell proliferation in TNBC cell lines. However, once palbociclib is removed during combined treatment with the PI3K inhibitors, this synergistic inhibition is lost, suggesting that continuous palbociclib treatment is required to maintain the inhibitory effects. In addition to cell cycle arrest (increased proportion in the G0 phase of the cell cycle), sequential treatment gave rise to greater inhibition of the Rb and PI3K/AKT/mTOR pathways that result in the induction of cell death compared to single and combination treatments.^102^ Furthermore, combination with alpelisib resulted in significantly reduced expression of GLUT‐1 and glucose uptake and consumption (25%) compared to palbociclib alone (15%).^102^ The inhibition of CDK4/6 by palbociclib has been implicated in the activation of AKT and the mTOR pathway resulting in activation of cell proliferation, conferring resistance in Rb‐positive BC.^129^, ^130^ Thus, further investigations in preclinical models of TNBC disease are required to pave the way for the clinical utility of dual inhibition of the CDK4/6 and mTOR/PI3K pathways for TNBC patients.

### 5.2 Novel CDK7 inhibitors—Overcoming resistance to CDK4/6 inhibitors

As part of the mechanism of resistance to CDK4/6 inhibition, other cell cycle regulators may be activated to compensate for CDK4/6 activity. One of these is CDK7, which acts as a CDK‐activating kinase (CAK) to regulate the G2/M phase by establishing CDK1 and CDK2 activity. In addition, CDK7 has been shown to maintain CDK4/6 in an active state.^131^ In addition to cell cycle regulation, CDK7 is a component of the basal transcriptional factor TFIIH that0 phosphorylates RNA polymerase 2 (PolII), resulting in transcription of genes required for progression through the cell cycle.^132^ CDK7 thus makes an interesting potential target for anti‐cancer therapies and the agent ICEC0942 is shown to be a potent CDK7‐specific inhibitor in both ER‐positive and TNBC cell lines.^133^ ICEC0942 elicited strong inhibitory effects on cell proliferation and promoted apoptosis through reduced levels of PolII in both the ER‐positive (MCF7 and T47D) and TNBC (MDA‐MB‐231 and MDA‐MB‐468) cells. A study investigated that the CDK7 inhibitor THZ1 reported that TNBC cell lines exhibited greater sensitivity to CDK7 inhibition than ER‐positive cells, as seen by potent inhibition of cell proliferation; however, phospho‐PolII status was equally affected in both BC subtypes.^134^ In contrast, cell death was elicited in TNBC cells by the induction of PARP and caspase 3 cleavage, but not in ER‐positive BC cells. In vivo studies showed that THZ2 (an improved analogue of THZ1) significantly reduced the rate of tumor growth in a TNBC xenograft mouse model compared to the vehicle‐treated mice.^134^ Interestingly, the knock‐down of CDK7 by CRISPR/Cas9 preferentially suppressed TNBC cell growth, whereas ER‐positive BC cells were unaffected, demonstrating the high dependency of CDK7 in TNBC. These studies provide an interesting foundation for the testing the combination of CDK4/6 and CDK7 inhibitors in for patients with TNBC, where otherwise limited options are available. The combination of such inhibitors will likely give rise to various adverse effects and toxicities, because a number of pathways are targeted. It is therefore imperative to assess the off‐target effects (for example, the impact on hematological cells) and safety of the drugs in combination or sequence.

## 6 SUMMARY

Cytotoxic chemotherapy continues to serve as the backbone therapy for TNBC; however, prognosis remains poor for TNBC patients. Neoadjuvant chemotherapy results in greater pCR in TNBC than non‐TNBC, with recent trials showing improvements in OS and DFS. Furthermore, incremental benefits are being observed in the neoadjuvant TNBC setting with the addition of platinum‐based chemotherapy and more recently PD‐L1 inhibitors. Additionally, although utilizing polychemotherapy in a dose dense manner seems to be the most effective approach in the adjuvant setting, optimized regimens are yet to be determined particularly for mTNBC. Additionally, following the success of CDK4/6 inhibitors in ER‐positive disease, the efforts to identify novel therapeutic approaches to TNBC continue. Preclinical studies support CDK4/6 inhibition as a plausible approach in TNBC in combination with current chemotherapies, particularly in sequential treatments playing a role in sensitizing cells providing further benefit compared to chemotherapy alone. With results from ongoing CDK4/6 inhibitor trials in TNBC, it may be possible to identify timings for the administration of these inhibitors in parallel with chemotherapeutic or novel agents in (neo)adjuvant treatments. With more recent cases of resistance to CDK4/6 inhibitors, further data from these trials will also provide valuable resources to identify potential biomarkers to help determine subgroups of patients with TNBC most likely to benefit from CDK4/6 inhibitors. Importantly, TNBC is a heterogeneous disease characterized by molecular phenotypes and signaling pathways. Thus, the profiling of these different subtypes is imperative for the success of personalized therapies for TNBC patients while avoiding exposure to toxicities for those who may not benefit.

## CONFLICT OF INTEREST

All authors declare no conflicts of interests.

## FUNDING

Not applicable.

## ETHICS APPROVAL

Not applicable.

## AVAILABILITY OF DATA

Not applicable.

## CONTRIBUTIONS

All authors contributed to the writing, review and revision of the manuscript.
